# P-630. Seasonal Influenza Vaccination and Household Contact Susceptibility to Influenza and Symptomatic Infection Outcomes: a Case-Ascertained Household Transmission Study, United States, 2023-2024

**DOI:** 10.1093/ofid/ofaf695.843

**Published:** 2026-01-11

**Authors:** Henry Fremont, Carlos G Grijalva, Melissa Stockwell, Stacey L House, Rachel M Presti, Stephanie A Fritz, Elizabeth B White, Keipp Talbot, Son H McLaren, Ellen Sano, Celibell Vargas, Anny L Diaz Perez, Jonathan Schmitz, Yuwei Zhu, Theresa A Scott, Caroline O’Neil, Francesca Yerbic, Bijal Parikh, Andrew Atkinson, Jessica E Biddle, Emily A McNair, Samantha G Dean, Lydia Bristol, Alexandra Mellis, Jennie H Kwon

**Affiliations:** Washington University in St. Louis, St. Louis, Missouri; Vanderbilt University Medical Center, Nashville, Tennessee; Columbia University Irving Medical Center, New York City, NY; Washington University School of Medicine, St. Louis, Missouri; Washington University, Saint Louis, MO; Washington University School of Medicine, St. Louis, Missouri; Influenza Division, Centers for Disease Control and Prevention, Atlanta, Georgia; Vanderbilt University Medical Center, Nashville, Tennessee; Columbia University Irving Medical Center, New York City, NY; Columbia University Irving Medical Center, New York City, NY; Columbia University Irving Medical Center, New York City, NY; Columbia University Irving Medical Center, New York City, NY; Vanderbilt University Medical Center, Nashville, Tennessee; VUMC, NASHVILLE, Tennessee; Vanderbilt University Medical Center, Nashville, Tennessee; Washington University, Saint Louis, MO; Washington University in St. Louis, St. Louis, Missouri; Washington University School of Medicine, St. Louis, Missouri; Washington University School of Medicine, St. Louis, Missouri; Centers for Disease Control and Prevention, Atlanta, Georgia; Centers for Disease Control and Prevention, Atlanta, Georgia; CDC, Atlanta, Georgia; Centers for Disease Control and Prevention, Atlanta, Georgia; Centers for Disease Control and Prevention, Atlanta, Georgia; Northwestern University , Chicago , IL

## Abstract

**Background:**

The goal of this study was to determine the impact of vaccination on the risk of influenza virus infection and symptomatic illness through a multi-center household transmission framework.

**Figure:**
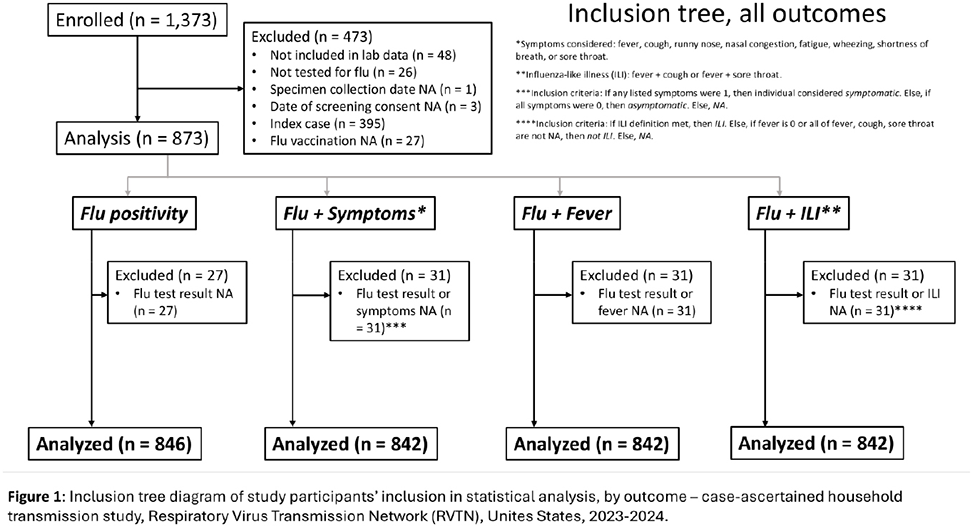

**Figure:**
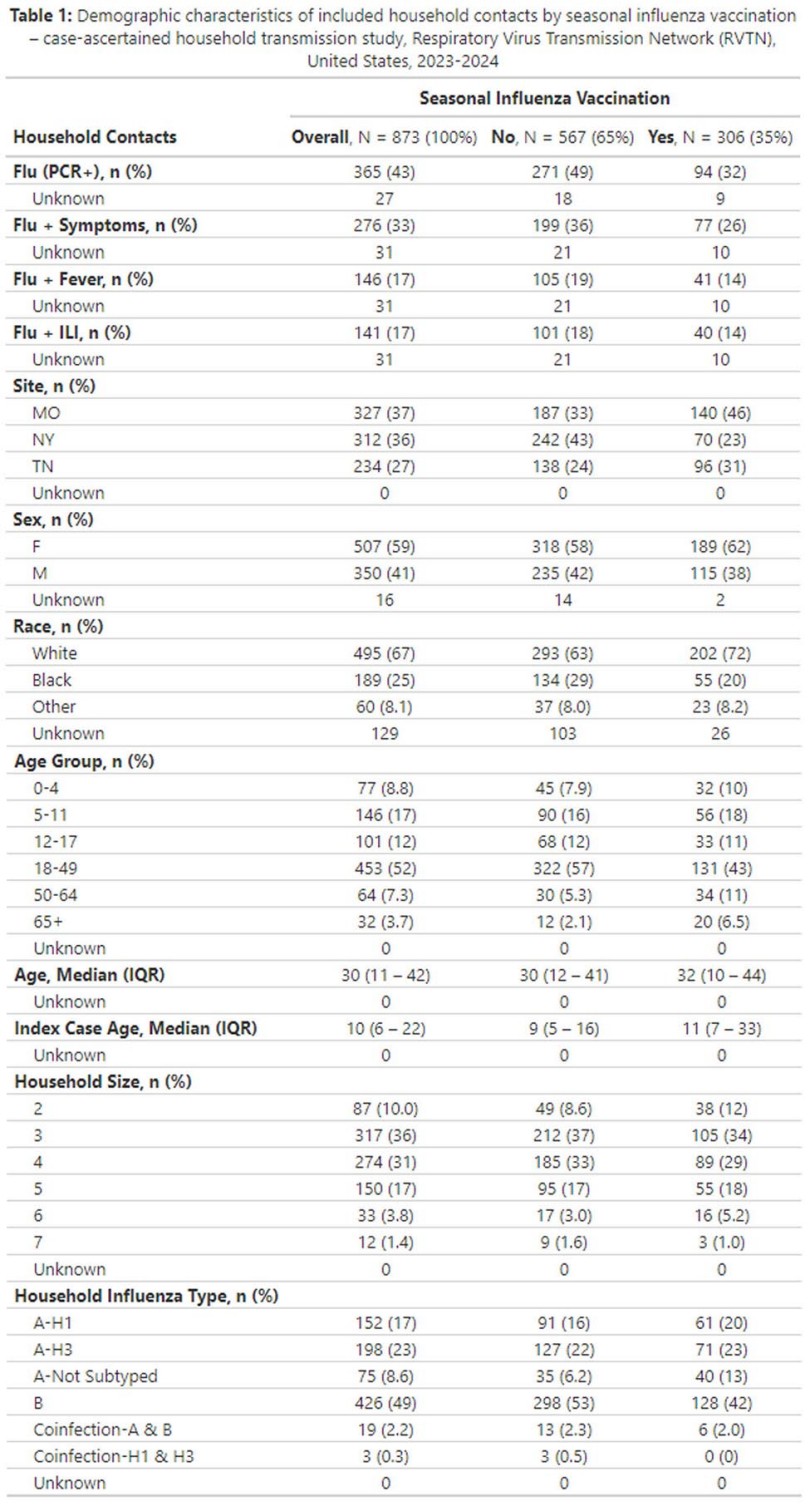

**Methods:**

The Respiratory Virus Transmission Network (RVTN) Flu is a case-ascertained household transmission study where influenza virus-infected index cases and their household contacts are recruited and enrolled within 6 days of index case symptom onset at 3 US sites (MO, NY, TN). Demographics and influenza vaccination status are collected from each household member at enrollment, along with a retrospective diary of symptoms; vaccination was defined as receipt of seasonal dose 14 days before index onset and confirmed via registry and medical record data. After enrollment, all participants complete daily follow-up for up to 7 days, self-collecting a nasal swab (Aptima, Hologic) and completing symptom diaries. Swabs are tested for influenza via RT-PCR (Hologic Fusion).

We examined the effectiveness of seasonal influenza vaccination in household contacts during the 2023-24 influenza season against infection and 3 different categories of symptomatic influenza: any symptoms, fever, and influenza-like illness (ILI). Figure 1 details inclusion criteria and definitions.

Logistic regression models with a generalized estimating equation framework were fit to estimate the association between seasonal influenza vaccination and infections in household contacts, accounting for household clustering and relevant covariates. Vaccine effectiveness was interpreted as 1-adjusted odds ratio (OR).

**Figure:**
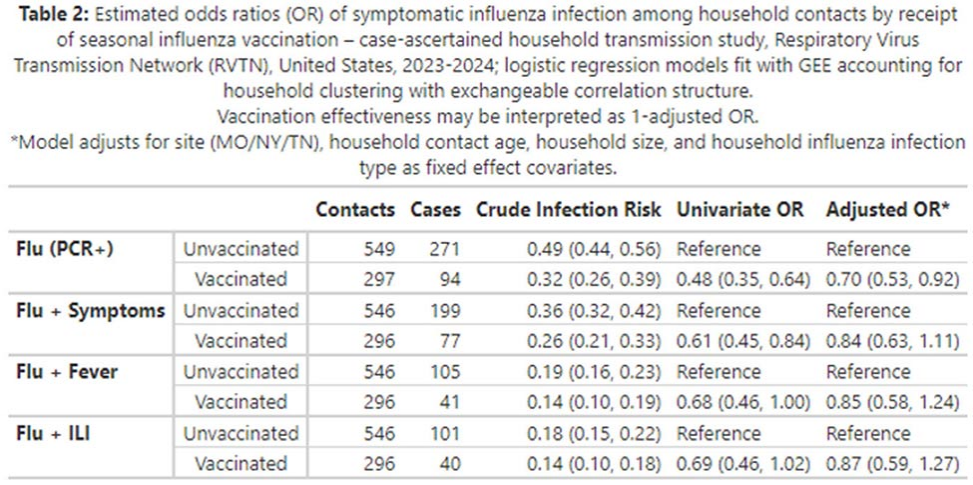

**Figure:**
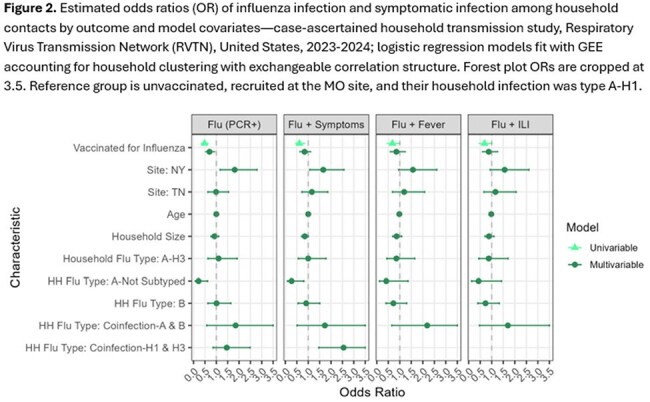

**Results:**

A total of 873 household contacts from 387 households were included (Table 1). Overall, 35% of contacts were vaccinated.

Influenza vaccination was associated with reduced odds of influenza virus infection when adjusted for site, age, household size, and household influenza virus infection type/subtype (Estimated Vaccine Effectiveness 0.32 (95% CI 0.05-0.59); Table 2, Figure 2). Vaccination was associated with lower adjusted odds of symptomatic influenza, although these results were not statistically significant.

**Conclusion:**

Current season influenza vaccination was associated with a reduced likelihood of testing positive for influenza after exposure to influenza in the household.

**Disclosures:**

Carlos G. Grijalva, MD MPH, AHRQ: Grant/Research Support|CDC: Grant/Research Support|GSK: Advisor/Consultant|Merck: Advisor/Consultant|NIH: Grant/Research Support|Syneos Health: Grant/Research Support Melissa Stockwell, MD MPH, CDC: Grant/Research Support Stacey L. House, MD, PhD, Abbott: Grant/Research Support|Alfa Scientific: Grant/Research Support|Beckman Coulter: Grant/Research Support|Biomerieux: Grant/Research Support|Cepheid: Grant/Research Support|CorDx: Grant/Research Support|Dompe: Grant/Research Support|Healgen: Grant/Research Support|Hologic: Grant/Research Support|InBios: Grant/Research Support|Inflammatix: Grant/Research Support|MeMed Diagnostics: Grant/Research Support|Meridian: Grant/Research Support|Nuclein: Grant/Research Support|Roche: Grant/Research Support|Seegene: Grant/Research Support|Sysmex: Grant/Research Support|Werfen: Grant/Research Support|Wondfo: Grant/Research Support Bijal Parikh, MD, PhD, Cepheid: Honoraria

